# De-Novo Transcriptome Sequencing of a Normalized cDNA Pool from Influenza Infected Ferrets

**DOI:** 10.1371/journal.pone.0037104

**Published:** 2012-05-11

**Authors:** Jeremy V. Camp, Thomas L. Svensson, Alexis McBrayer, Colleen B. Jonsson, Peter Liljeström, Carl E. Bruder

**Affiliations:** 1 Center for Predictive Medicine for Biodefense and Emerging Infectious Disease, University of Louisville, Louisville, Kentucky, United States of America; 2 Science for Life Laboratory, Stockholm, Solna, Sweden; 3 Southern Research Institute, Birmingham, Alabama, United States of America; 4 Department of Microbiology, Tumor and Cell Biology, Karolinska Institutet, Stockholm, Sweden; Centre of Influenza Research, The University of Hong Kong, Hong Kong

## Abstract

The ferret is commonly used as a model for studies of infectious diseases. The genomic sequence of this animal model is not yet characterized, and only a limited number of fully annotated cDNAs are currently available in GenBank. The majority of genes involved in innate or adaptive immune response are still lacking, restricting molecular genetic analysis of host response in the ferret model. To enable de novo identification of transcriptionally active ferret genes in response to infection, we performed de-novo transcriptome sequencing of animals infected with H1N1 A/California/07/2009. We also included splenocytes induced with bacterial lipopolysaccharide to allow for identification of transcripts specifically induced by Gram-negative bacteria. We pooled and normalized the cDNA library in order to delimit the risk of sequencing only highly expressed genes. While normalization of the cDNA library removes the possibility of assessing expression changes between individual animals, it has been shown to increase identification of low abundant transcripts. In this study, we identified more than 19000 partial ferret transcripts, including more than 1000 gene orthologs known to be involved in the innate and the adaptive immune response.

## Introduction

Recent advances in DNA sequencing have drastically increased the sequencing throughput relative to traditional Sanger based approaches [1]–[3]. Second-generation DNA sequencing technology enables more cost-efficient genomic analysis and, due to their highly parallel nature, allows for applications in transcriptomics and epigenomics. De-novo transcriptome sequencing of normalized cDNA libraries constructed from tissues or cells allows for full or partial determination of transcribed genes, and is a fast and efficient way to obtain sequence information for organisms where there is no or little sequence available in the public sequence repositories [4]–[8]. Normalization delimits the risk of sequencing only high abundant transcripts, and thus enhances the chance of de-novo identification of low expressed transcripts that may have escaped detection other vice. A consequence of this procedure is that determination of individual expression profiles is not possible. The sequence information obtained from de-novo transcriptome sequencing does open up for development of species specific microarrays and taq man assays, and can be used as reference transcriptome for when determining expression profiles using RNA-sequencing (RNA-seq) [8]–[12].

The ferret has become an important model for studies of infectious diseases, such as influenza virus, severe acute respiratory syndrome coronavirus and canine distemper virus [13]–[20]. In addition, the ferret has recently been used to study the cortical development [21], [22], as well as study regenerative endodontics [23]. Until recently, only a limited number of partial or full length cDNAs were present in the GenBank, limiting the molecular genetic assays available for analysis. In a recent study, we performed de novo sequencing using highly parallel sequencing of normalized cDNA derived from blood, liver, lung, spleen and brain from healthy ferrets. In that study, approximately 16000 partial ferret transcripts were identified and used to develop a ferret specific microarray [8]. Although the generation of these transcripts increased the number of sequences available for molecular genetics analysis of the ferret model, many genes known to be involved in the response to infection were not identified, still hampering comprehensive analyses of genetic alterations in the ferret in response to pathogens.

Here we report the utilization of massively parallel sequencing using the Roche GS-FLX titanium platform to further increase the number of annotated expressed sequence tags for the ferret. To increase the detection ability of genes known to be induced by viral or bacterial pathogens, the cDNA library was constructed using tissues from animals infected with influenza virus as well as ferret splenocytes induced with bacterial lipopolysaccharide (LPS) to allow for identification of Gram-negative pattern recognition receptors such as Toll-like receptor 4. We have identified more than 19000 partial ferret transcripts, of which more than 1000 show high sequence resemblance to gene orthologs involved in the innate and adaptive immune response.

## Results

The massively parallel sequencing of the pooled and normalized cDNA library generated 1’130’000 reads, corresponding to approximately 300 Mbp. These reads were assembled using iAssembler and subjected to annotation analysis using megablast as well as normal blast algorithms against the Refseq RNA, RefSeq Protein, Refseq Human RNA and Refseq Human Protein databases. Using Megablast, 51076 contigs showed alignment to proteins or RNA sequences in the Refseq RNA or protein databases. These corresponded to 24972 individual accessions numbers in the RefSeq RNA or protein database. When blasted against the human genes or proteins, 20767 contigs were aligning 9795 individual human orthologs (using an e^norm^ value cut off of >0.8). These orthologs were used for subsequent analysis to identify overrepresented biological clusters. As the cDNA library was constructed using several tissues, the fact that the most overrepresented biological functions describe general cellular functions, such as cellular process (GO:0009987), metabolic process (GO:0008125) and gene expression (GO:0010467) was not surprising. As we infected the animals prior to the harvest of the tissues, we expected an enrichment of genes known to be induced during different stages of early infection in this data set when compared to the data previously generated. Using the Gene Ontology database, we found that 741 (53%) of the ferret gene orthologs overlapped with genes within the most ancestral GO-term representing the immune system process (GO:002376). Similar comparisons using the EST data set we have reported previously, indicated that 421 ferret orthologs were overlapping with the genes in GO:0002376. The approach using a normalized cDNA library from a pool of tissues from infected animals as well as LPS induced ferret splenocytes thus proved to be a valid way to identify ferret gene orthologs involved in the immune response.

**Figure 1:**
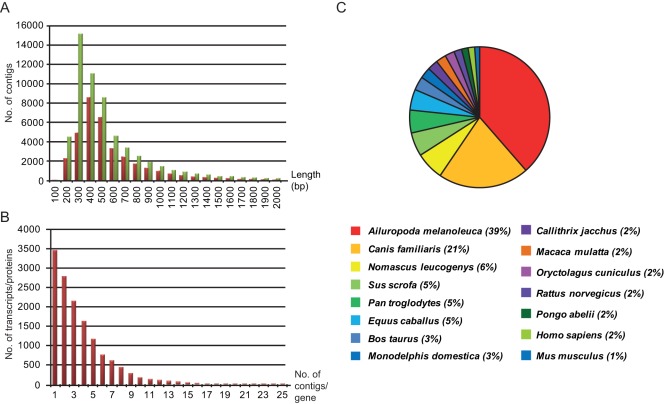
The histogram in panel A shows the distribution of contigs length of the sequence assemblies. The red bars indicate the length of the contigs from the assembly using the sequences from the cDNA library from infected tissues. The green bars show the contigs lengths for the combined assembly, built from these sequences as well as the ESTs previously reported [8]. The histogram in panel B illustrates the number genes that were represented by one or multiple non-overlapping of contigs in the combined assembly. Panel C shows the distribution of species extracted using the best hit (lowest blast E-value) from the Refseq Protein blast analysis of the combined assembly.

To maximize the de-novo identification of ferret gene orthologs, and increase the read lengths of the contigs, we made an assembly with the ESTs generated in this study and those we have previously have reported [8]. Subsequent blast alignment generated 58’891 contigs with hits to proteins or RNA sequences when blasted against the Refseq RNA and RefSeq Protein databases (corresponding to 32’496 different proteins/transcripts). As the Refseq RNA and Protein databases contain all known orthologs of genes, counting the only the number of individual hits to the entries in these databases may lead to an overestimation of the number of actual genes identified. This is exemplified by contigs aligned to different orthologs of the aminoadipate-semialdehyde dehydrogenase gene (*AASDH*), where some contigs show the highest overlap to the *AASDH* gene from giant panda, whereas other contigs are more similar to the dog ortholog. To improve the estimate of the number of genes identified, we extracted the gene symbol from the annotations and counted the number of individual gene symbols rather than the accession number from the RefSeq blast hit. This resulted in 19’342 partially sequenced genes (Tables S1 and S2). A large number of these genes were represented by two or more non overlapping contigs, which may be indicative of different splice variants (Fig. 1).

Five thousand thirty nine of the 19’342 transcripts were aligned to genes staring with LOC followed by the geneID. For such transcripts, no published gene symbol is available nor has an ortholog been determined yet. Gene annotation processes are continuing at the NCBI, and the actual number of individual genes identified in this data set may be altered. Investigating the best hit for each contig from the Refseq protein blast analysis showed that most hits were gene orthologs of the giant panda (*A. melanoleuca*, 14’816 contigs) and the domestic dog (*C. familiaris*, 8038 contigs) (Fig. 1). Both of these species, as well as the ferret, are part of Caniformia, a suborder within the order Carnivora. More than 7000 contigs showed high similarity to LINE-1 reverse transcriptase and were removed from functional analysis.

Using the human protein and RNA databases for the blast analysis resulted in 52’510 contigs being aligned to human proteins or RNAs (corresponding to 13’697 different human transcripts or proteins). As we expected these genes to represent the a large amount of the expressed ferret genes, it was not surprising that the most overrepresented gene-annotation categories from the enrichment analysis were of a more general type, such as “cellular process”, “metabolic process”, “macromolecule biosynthetic process” and “protein localization”. When investigating the overlap with the genes associated the GO-term representing the immune system process (GO:002376), we found that the combined sequence assembly contained full or partial transcripts corresponding to almost 80% (1078 of 1390 genes) of the human gene orthologs associated with this GO-term (Table S3).

## Discussion

In this study, we have continued our effort in identifying ferret transcript sequences. As we and others have previously shown, de-novo sequencing of normalized cDNA libraries is a straight forward approach to increase the sequence information of species with little or no previous genetic information available [6], [10], [24]–[26]. In our previous study, we sequenced pooled total RNA from tissues obtained from healthy adult ferrets, and as a consequence, the identification of transcripts induced by various infections was not complete [8]. Since the ferret is commonly used to study infection, especially for influenza, we isolated tissues from animals infected with H1N1 A/California/07/2009. In order not to overlook the genes specifically induced in response to bacterial infection, we also included total RNA from ferret splenocytes induced by LPS, a major component of the cell wall of gram-negative bacteria. We selected the Roche GS-FLX titanium platform for this study, as this platform allows for longer, albeit fewer, sequence reads than other comparable platforms. The longer reads allow for accurate assemblies with fewer gaps, longer contigs and better assignment of repeat regions. In addition, we normalized the cDNA library prior to the sequencing analysis, as highly abundant transcripts may impede de novo identification of low-expressed or tissue specific genes [27], [28]. Pooling and normalization of the total RNA followed by GS-FLX titanium sequencing provided a comprehensively annotated ferret reference transcriptome. Albeit this approach removed the ability to determine the actual gene expression levels between our samples, the ferret transcriptome generated here will be an essential tool to map ferret gene expression changes assessed by RNA-sequencing.

We performed two parallel lines of assembly, one where the sequences generated from the normalized cDNA library constructed here, and one where we also included the sequences we had generated previously. The reasoning behind the two separated assemblies was that we wanted to assure that the detection of gene orthologs involved in the immune response was enriched. Comparisons to the gene ontology database did show that an increased number of different transcripts were increased in this dataset. Several of these ferret orthologous sequences have not been previously known, for instance, the ferret sequence information for the C-C chemokine receptor type 5 (*CCR5*), one of the co-receptors for the HIV virus, was not previously present in GenBank [29], [30]. We identified a partial transcript of more than 900 base pairs that aligned to the *CCR5* ortholog genes in panda, dog, horse, pig and human. Another example of de-novo identification of a gene important for immune response was the ferret gene ortholog of the T-cell surface glycoprotein CD5. CD5 is a transmembrane protein expressed constitutively on the surface of T cells and a subset of B cells [31]. We identified a partial transcript of more than 1000 base pairs showing high resemblance to the *CD5* ortholog genes in several other species, including panda, dog, horse and cow. Other genes of interest that were identified where no ferret sequence information previously has been known are the forkhead box P3 gene (*FOXP3),* the genes encoding the intercellular adhesion molecules 3 (*ICAM3)*, the interferon regulatory factor 8 gene (*IRF8*) and the interleukin-1 receptor-associated kinase 2 gene (*IRAK2*). The increased number of genes involved in immune response is in agreement with a host gene response to pathogens, and we show that generation of an RNA pool from infected animals is a straight forward path to identify immune response genes in non-standard animal models. The assembly where also the ESTs we previously have identified were included was done to maximize the de-novo identification of ferret gene orthologs, as well as increase the length of these transcripts. This analyzed in identification of more than 19000 partially sequenced genes, of which more than 1000 transcripts showed high homology to gene orthologs known to be involved in the immune response. We provide here an extensive analysis of the ferret transcriptome, promoting development of additional molecular tools for this increasingly important animal model.

## Materials and Methods

All in-vivo procedures were conducted in accordance with the Animal Welfare Act and the CDC-NIH Biosafety in Microbiological and Biomedical Laboratories and were approved by the Southern Research Institutional Biosafety Committee and Institutional Animal Care and Use Committee (ACUP protocol #08-05-031B). The Animal experiments were performed in the AAALAC-accredited ABSL-2 facilities at Southern Research Institute, Birmingham, AL. Four ferrets, two males and two females were intranasaly infected with 10^4^ TCID50/ml influenza H1N1 A/California/07/2009. Viral titers in nasal swabs were analyzed by establishing the median tissue culture infective dose in Madin-Darby canine kidney cells according to our previously published protocols [32]. Analysis of nasal swabs is a straight forward method to analyze presence of replicating virus during influenza infection ([33] and our unpublished results). One male and one female were euthanized on days three and six post-infection to increase chance of identifying transcripts with specific temporal expression patterns. Tissues were collected and snap frozen in liquid nitrogen. We collected cranial and caudal lung, heart, bone marrow from the femur, lymph node, spleen and blood.

The tissues were homogenized in TRIzol and RNA was extracted according to the protocol supplied by the manufacturer (Invitrogen). RNA from blood was extracted using the PAX gene kit from Qiagen, according to the supplier’s instructions. Spleens from naïve, healthy male ferrets were collected over ice into Dulbecco’s phosphate buffered saline (Gibco). Splenocytes were released by mechanical disruption and the cells were passed through 40 µm mesh strainers. The cells were pelleted by centrifugation. Erythrocytes were lysed in ammonium chloride-based lysis buffer (ACK Lysis Buffer, Lonza) for 5 minutes, followed by washing in heat-inactivated fetal calf serum (FBS, Gibco). Splenocytes were washed and the concentration was adjusted to 2×10^6^ cells/ml in RPMI-1640 media with 5% FBS, 1% Penicillin/Streptomycin, and 200 µM L-glutamine. Lipopolysaccharide from E. coli O111:B4 (Sigma-Aldrich) was added to the media at a concentration of 200 ng/ml and cells were incubated overnight (10 hours) at 37°C with 5% CO_2_ and saturated humidity in a cell culture flask. Activation was confirmed under an inverted light microscope, the cells were pelleted and lysed in TRIzol. After quality assessment of the RNA using the BioRad Experion RNA StdSens analysis kit, the RNA samples were pooled to ensure sequence diversity. This RNA pool was reverse-transcribed to cDNA that was normalized prior to sequencing to avoid sequencing of abundantly expressed transcripts. Preparation of the normalized cDNA library was then done as previously published [8] and sequenced using 454 Sequencing on the Genome Sequencer FLX with GS FLX Titanium series reagents according to the manufacturers protocols (Roche).

Assembly of the sequencing reads was done with iAssembler (version: v1.3) using default settings [34]. This software is a standalone package designed to accurately assemble longer next generation reads generated by the Roche-454 sequencing technology into contigs. The pipeline employs an iterative assembly strategy with automated error corrections of miss-assemblies to increase the accuracy of the EST assembly [34]. Two separate assemblies were built, using the sequences from the cDNA library constructed here (TSA accession numbers JR778296 - JR808535), and a combined assembly of these sequences and those that we had generated previously [8]. This assembly was done to maximize the chances of de novo identification of ferret gene orthologs. The assemblies were then blasted against the RefSeq RNA and Protein databases, as well as the RefSeq RNA and Protein databases specific for humans. We used both a more stringent analysis (MegaBlast) and regular blast to annotate the contigs. Extraction of annotation data was done using a TCL script called tcl_blast_parser, which provides a matrix file containing info about the primary blast hits, including normalized expectation values for the hits (e^norm^, -log(Exp)/100), the number of perfect matches, alignment length, frame of translation etc. The contigs that aligned with human nucleotide and/or protein sequences using the MegaBlast algorithm are shown in Table S1. Contigs without human orthologous sequences or contigs aligning with non-transcribed genes and contigs exclusively aligned using the less stringent blastn algorithm are found in Table S2. The searches against the human databases enabled functional analysis using the database for annotation, visualization and integrated discovery (DAVID, version 6.7) as well as the BiNGO Cytoscape (version 2.44) plug-in to assess overrepresentation of gene ontologies (GO) [35]–[37]. The settings used for the BiNGO analysis were: Hypergeometric testing followed by Benjamin & Hochberg FDR correction using a P-value 0.05 for Biological_Process in *Homo sapiens*. http://publications.scilifelab.se/svensson_lt/ferret_transcriptome.

## Supporting Information

Table S1 **Annotation table for ferret ESTs with human sequence homology.** Contigs from the combined assembly of cDNA sequences indentified here and the ESTs previously reported [8] that showed homology to human RNA or protein sequences using the “megablast” algorithm in BLAST. “Similar to Human Gene” and “Human Refseq acc no.” indicate the Human Gene Symbol and the accession number from the Human RNA or Human Protein database. “RefSeq RNA_acc” points to the accession number from the RefSeq RNA blast analysis, using all RNA in the GenBank database. The nucleotide sequence for each contigs is displayed in the column denoted “Sequence.”
(XLSX)

Table S2 **Annotation table for ferret ESTs without human sequence homology, ESTs with homologies detected exclusively by blastn and ESTs with homology to untranscribed human gene orthologs.** The “no_human_hits” worksheet contains the lists the contigs from the combined assembly that did not show homology to human RNA or protein sequences using the “megablast” algorithm in BLAST, but were aligned with RNA sequences from the RefSeq RNA database. The “RefSeq Gene from refseq RNA” indicates the gene name for the orthologous sequence, derived from the description line of the blast hit, “RefSeq RNA_acc” indicates the accession number of the homologous sequence. The “blastn” spreadsheet lists the contigs that showed alignment when the less stringent blastn algorithm was used, and the “hits_to_non_transcr_hum_genes” shows the contigs that showed the best alignment to known non-transcribed human orthologs. Here, “Similar to Human Gene” and “Human Refseq acc no.” indicate the Human Gene Symbol and the accession number from the Human RNA or Human Protein database. “RefSeq RNA_acc” points to the accession number from the RefSeq RNA blast analysis, using all RNA in the GenBank database. The nucleotide sequence for each contigs is displayed in the column denoted “Sequence.”
(XLSX)

Table S3 **Ferret sequence of genes in immune system process (GO:002376).** “Gene symbol,” “Description“ and “Human Refseq acc no.” indicate the Human Gene Symbol, the protein description and the accession number from the Human RNA or Human Protein database. “RefSeq RNA Acc. no.” points to the accession number from the RefSeq RNA blast analysis, using all RNA in the GenBank database. “Contig” indicates the ferret contig aligning with the corresponding human gene ortholog. The nucleotide sequence for each contigs is displayed in the column denoted “Ferret Sequence.”
(XLSX)
